# Clinical features and outcomes of fusion gene defined adult Ph-negative B-cell precursor acute lymphoblastic leukemia patients: A single institutional report

**DOI:** 10.17305/bjbms.2022.7851

**Published:** 2023-03-16

**Authors:** Kai Sun, Jun Wang, Wen-Min Chen, Nan Xu, Ling-Yu Long, Xu Wang, Hao Jiang, Qian Jiang, Xiao-Jun Huang, Ya-Zhen Qin

**Affiliations:** 1 Peking University People’s Hospital, Peking University Institute of Hematology, National Clinical Research Center for Hematologic Disease, Beijing Key Laboratory of Hematopoietic Stem Cell Transplantation, Beijing, China

**Keywords:** B-cell precursor acute lymphoblastic leukemia (BCP-ALL), Ph-negative, fusion genes, risk stratification, relapse-free survival (RFS)

## Abstract

More clinical studies are needed to clarify the risk stratification by the integration of all fusion genes in adult B-cell precursor acute lymphoblastic leukemia (BCP-ALL). A total of 320 consecutive adult Ph-negative BCP-ALL patients who had been tested classical fusions (*KMT2A* rearrangement and *TCF3-PBX1*) at diagnosis were further retrospectively screened novel fusion genes (*Ph-like*, *ZNF384,* and *MEF2D* fusions) by multiplex real-time quantitative PCR (RQ-PCR). Classical fusions were identified in 12.5% of patients, while 4.4%, 17.2%, and 3.8% of patients were identified *Ph-like*, *ZNF384,* and *MEF2D* fusions, respectively. 1-course CR rate, relapse-free survival (RFS), and overall survival (OS) rates tended to show or showed statistically significant differences among fusion-defined subgroups (*P* ═ 0.084, <0.001, and 0.0093, respectively). Based on individual outcomes, patients with *KMT2A* rearrangement, *TCF3-PBX1*, *Ph-like,* and *MEF2D* fusions were classified into fusion-defined high-risk group (*n* ═ 66, 20.6%). High-risk group had significantly lower 3-year RFS and 3-year OS rates than standard-risk group (*P* < 0.001 and ═ 0.0022) and was an independent adverse prognostic factor for RFS in the entire cohort (*P* < 0.001). In conclusion, the spectrum of fusion genes in the current Chinese cohort was distinct from that in reports from western countries. Detection of fusion genes improved risk stratification in adult Ph-negative BCP-ALL patients.

## Introduction

B-cell precursor acute lymphoblastic leukemia (BCP-ALL) is a kind of disease with strong molecular heterogeneity, which is closely related to the formation of fusion genes caused by chromosomal rearrangement [1]. The 2016 revision to World Health Organization classification of B-lymphoblastic leukemia/lymphoma has classified *BCR-ABL1*, *KMT2A* rearrangement, *ETV6-RUNX1*, *IL3-IGH*, *TCF3-PBX1,* and *Ph-like* fusions as separate BCP-ALL subtypes [2]. In addition, insight into the identification of novel genetic subtypes has been deepening over the past decade. Novel genes and related fusions were increasingly found in BCP-ALL, such as *ZNF384* fusions [3], *MEF2D* fusions [4], *DUX4* fusions [5, 6], and more various *Ph-like*-related fusions [7].

Currently, remission rates of acute lymphoblastic leukemia (ALL) after the use of standard protocols in adults patients have reached 60%–92% [8], but 5-year overall survival (OS) rates remain less than 45% predominantly due to higher relapse rates [9, 10]. Growing research has confirmed that relapse and OS are closely related to fusion genes [4, 6, 10–13]. Therefore, it is of great clinical significance to identify high-risk cases based on fusion gene detection and perform risk stratification as soon as possible for further treatment aiming at molecularly heterogeneous targets.

Compared to studies on the discovery of novel fusions, clinical cohort studies concerning treatment outcomes have been insufficient so far, and results were not fully consistent. In addition, although RNAseq technique has obvious advantages to discover novel fusion transcript, screening common fusion transcript by RQ-PCR is still a quick and practical method in clinical routine. In our previous study, we reported the incidence, characteristics, and prognostic role of *ZNF384* fusions in 242 patients [13]. As an extension and complement, in the present study, we continued to retrospectively perform TaqMan-based RQ-PCR to screen novel fusion genes (*Ph-like*, *ZNF384,* and *MEF2D* fusions) on the 10-year consecutive cases of our institute who had tested classical fusion genes at diagnosis, trying to investigate their incidence, clinical characteristics, and prognostic roles. Prognostic significance based on risk stratification was further explored, with a view to guiding the implementation of clinical protocols.

## Materials and methods

### Patients

Three hundred and twenty adult Ph-negative BCP-ALL patients who were consecutively diagnosed and received at least one cycle of induction chemotherapy at Peking University People’s Hospital from January 2009 to December 2020 were included. The diagnosis was based on bone marrow (BM) morphology, immunophenotyping, karyotyping, and molecular testing. The cutoff date for the last follow-up was November, 2021.

### Treatment

As we reported previously [14, 15], chemotherapy procedure consisted of induction, consolidation, and maintenance chemotherapy. CODP±L (cyclophosphamide, daunorubicin, vincristine, and prednisone, ±L-asparaginase) was used as the induction regimen. Patients who did not achieve CR after the first induction chemotherapy received reinduction chemotherapy MAE (mitoxantrone, cytarabine, and etoposide) or a modified hyper-CVAD (B) regimen (methotrexate and cytarabine). Modified hyper-CVAD (B) or hyper-CVAD (A) (cyclophosphamide, dexamethasone, vincristine and doxorubicin) was used after the year 2010, and CODP±L, high dose methotrexate, or CAM (cyclophosphamide, cytarabine, and mercaptopurine) was used before 2010 as the consolidation regimen. Methotrexate, cytarabine, and dexamethasone were applied to the prevention of CNSL via intrathecal administration. Patients achieving CR for the first time (CR1) were recommended to receive allogeneic hematopoietic stem cell transplantation (allo-HSCT) unless the donor was absent, the performance status was poor or patient refused. Detailed indications, conditioning regimen, donor selection, graft-versus-host disease prophylaxis, and the modified DLI regimen of allo-HSCT have been comprehensively described in our previous studies [16, 17].

### Detection of classical fusion transcript and *IKZF1* deletion

All patients were screened classical fusion transcript at diagnosis. Trizol Reagent and DNAzol (Invitrogen, CA, USA) were used to extract total RNA and DNA from BM samples collected at diagnosis, respectively. A High Capacity cDNA Reverse Transcription Kit (Applied Biosystems, Foster City, CA, USA) was used to synthesize cDNA. TaqMan-based real-time quantitative PCR (RQ-PCR) was used to detect fusion transcript of *BCR-ABL1*, *TCF3-PBX1*, and *KMT2A* (*KMT2A-AFF1*, *KMT2A-MLLT3*, *KMT2A-MLLT10*, *KMT2A-MLLT1*, *KMT2A-EPS15,* and *KMT2A-MLLT11*) as we described previously [18, 19]. *IKZF1* deletion was detected by RQ-PCR using DNA in 216 patients diagnosed after 2014 [20].

### Screening of novel fusion transcript

Patients who had no classical fusion transcript were retrospectively screened novel fusion transcripts on their BM samples collected at diagnosis. Multiplex TaqMan-based RQ-PCR was performed. Multiplex *ZNF384* fusions screened *EP300-ZNF384*, *CREBBP-ZNF384*, *TCF3-ZNF384*, *EWSR1-ZNF384,* and *TAF15-ZNF384* as we previously reported [13]. Multiplex *MEF2D* fusions screened *MEF2D-HNRNPUL1*, *MEF2D-BCL9*, *MEF2D-DAZAP1*, *MEF2D-HNRNPH1,* and *MEF2D-SS18*, and multiplex *Ph-like* fusions screened *ABL1*, *ABL2*, *PDGFRB*, *JAK2*, *CSF1R,* and *NTRK* fusions. If multiplex RQ-PCR showed exponential amplification, split-out RQ-PCR with primer and probe sets for the individual fusion transcript was performed to identify partner. All patients who were negative for the above fusions and the majority of patients with the above fusions were tested *P2RY8-CRLF2* by RQ-PCR. Partners of Ph-like fusions and all types of fusion transcripts used to design primers and probes came from previous reports [21, 22] and our unpublished RNAseq results. Primers and probes were designed using Primer 3 (v. 4.0).

### Minimal residual disease evaluation

The minimal residual disease (MRD) level of patients was detected by flow cytometry at remission and after each cycle of consolidation treatment as described in our previous report [15].

### Definitions

Complex karyotypes were defined as 5 or more chromosomal abnormalities in the absence of t(4;11), t(1;19), and t(14q32) or ploidy subgroups [23]. Medical Research Council (MRC) UKALLXII/Eastern Cooperative Oncology Group (ECOG) 2993 trial and Group for Research on Adult Acute Lymphoblastic Leukemia (GRAALL)-2003/2005 trial defined high-risk karyotype as (1) low hypodiploidy or near triploidy, (2) t(4;11) or 14q32 translocation, and (3) complex karyotypes [23, 24]. CR was defined as (1) the absence of extramedullary disease, (2) the presence of trilineage hematopoiesis which referred to neutrophils more than 1 × 10^9^ /L and platelets more than 100 × 10^9^/L, (3) less than 5% BM blast cells, and (4) no recurrence for four weeks [25]. Relapse referred to reappearance of more than 5% of blasts in peripheral blood or BM or emergence of extramedullary diseases [26]. Relapse-free survival (RFS) was measured from the date when CR was achieved to relapse, or to the last date of the BM morphology examination. OS was measured from diagnosis to death (regardless of the cause), and patients were queried at the date of last follow-up to determine whether they were still alive or censored on the date they were last known to be alive.

### Ethical statement

The study protocol was approved by the ethics committee of Peking University People’s Hospital and complied with the Declaration of Helsinki (2020PHB095).

### Statistical analysis

Kruskal–Wallis 1-way ANOVA for *k* samples and Mann–Whitney U test were performed on continuous variables. Fisher’s exact test was performed on categorical variables. Survival functions were estimated using the Kaplan–Meier method and compared using log-rank test. Variables associated with *P* values less than 0.05 in univariate analysis were entered in multivariable analysis performed by Cox model. *P* values less than 0.05 were considered statistically significant. SPSS 26.0 software package (SPSS Inc., Chicago, IL, USA) and GraphPad Prism 7 (GraphPad Software Inc., La Jolla, CA, USA) were used for data analysis.

## Results

### Patient outcomes

Out of 320 patients with Ph-negative BCP-ALL included in this study, 152 (47.5%) were male. The median age at diagnosis was 31 years (range, 15–65 years). The median follow-up period was 25.7 months (range, 1.5months–145.8 months) for the entire cohort and 37.8 months (range, 1.5 months–145.8 months) for the 193 (60.3%) patients who were still alive at the last follow-up. A total of 297 (92.8%) patients achieved CR after induction chemotherapy and 108 (36.4%) of them experienced a subsequent relapse with a median time of 7.1 months (range, 0.9–69.8 months). Of the 297 patients who achieved CR, 114 (38.4%) patients received chemotherapy alone, while 183 (61.6%) received chemotherapy followed by allo-HSCT (matched sibling donor, *n* ═ 47; haploidentical related donor, *n* ═ 132; matched unrelated donor, *n* ═ 4). The 3-year rates of RFS and OS in the patients who achieved CR were 60.5% (95% confidence interval (CI), 54.1–66.3%) and 63.9% (95% CI, 57.6–69.5%), respectively, and the 3-year OS rate in the entire cohort was 59.5% (95% CI, 53.5–65.1%).

### Expression pattern of *P2RY8-CRLF2* in Ph-negative BCP-ALL patients

**Figure 1.**
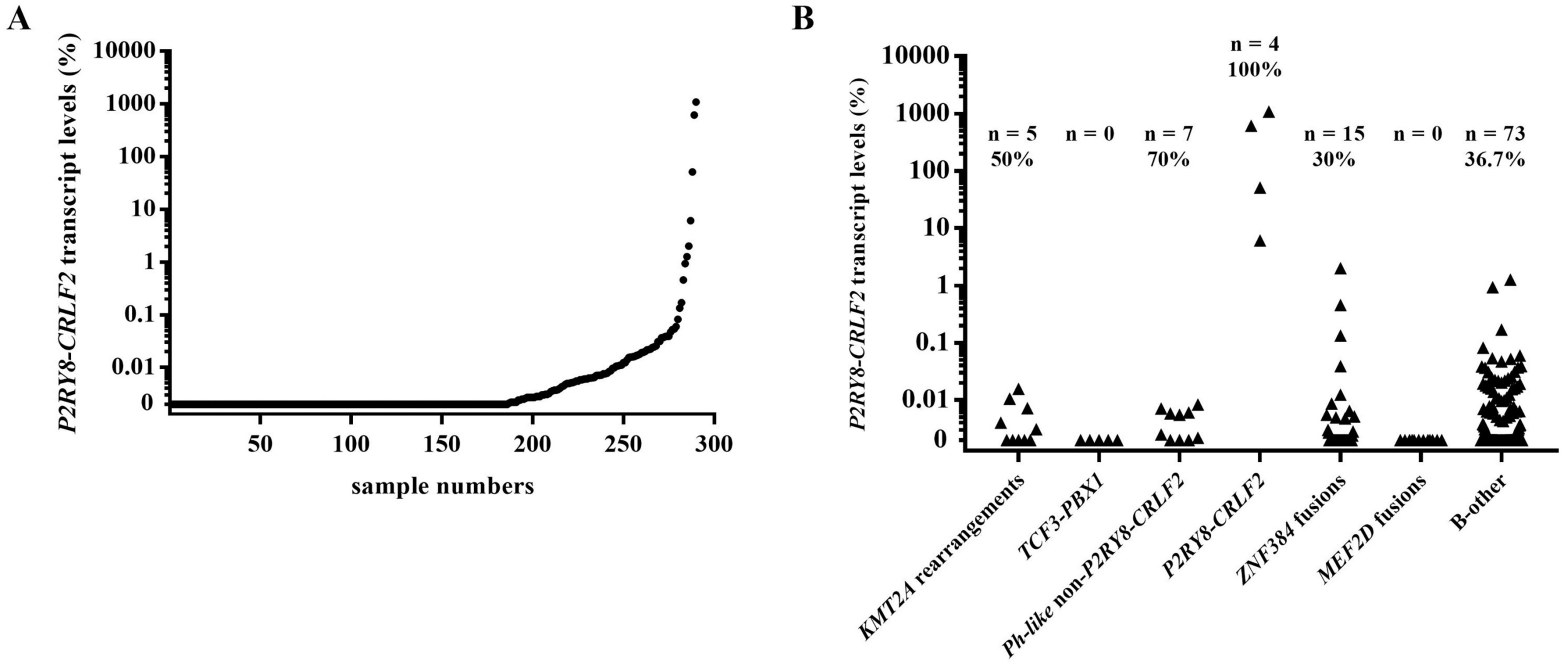
**The expression pattern of *P2RY8-CRLF2*.** (A) Expression pattern of *P2RY8-CRLF2* in 290 patients; (B) Expression pattern of *P2RY8-CRLF2* in patients grouped by fusion types with detectable numbers and proportion in fusion-defined groups shown above each graph.

Out of the 290 patients who were tested for *P2RY8-CRLF2*, 104 (35.9%) had a detectable transcript, ranging from 0.0007% to 1075.0%. The expression pattern of *P2RY8-CRLF2* in all 290 patients among the fusion-defined groups was shown in Figure 1. In 86 patients with fusion genes who were tested for *P2RY8-CRLF2*, 27 (31.4%) had detectable fusion transcripts (range: 0.0013%–2.01%) and the upper limit for *P2RY8-CRLF2* transcript was 2.01%. Therefore, 2.01% was set as the cut-off value to define *P2RY8-CRLF2* positive patients in the current study; and four patients were solely *P2RY8-CRLF2* positive and were referred to as *P2RY8-CRLF2* thereafter.

### Incidences of individual classical and novel fusion transcripts

**Figure 2.**
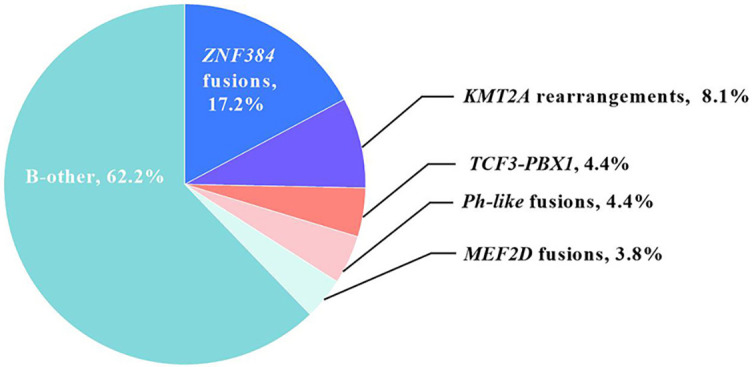
Distribution of fusion-defined patients.

Out of all 320 patients included, fusion transcripts were identified in 121 (37.8%) patients, and their distributions were as follows (Figure 2): 26 (8.1%) had *KMT2A* rearrangement (24 *KMT2A-AFF1*, 92.3%; 1 *KMT2A-EPS15*, 3.8%; and 1 *KMT2A-MLLT1*, 3.8%), and 14 (4.4%) had *TCF3-PBX1*. In addition, 55 (17.2%) patients had *ZNF384* fusion (43 *EP300-ZNF384*, 78.2%; 6 *CREBBP-ZNF384*, 10.9%; 3 *TCF3-ZNF384*, 5.5%; 2 *TAF15-ZNF384*, 3.6%; and 1 *EWSR1-ZNF384*, 1.8%); 12 (3.8%) had *MEF2D* fusion (7 *MEF2D-HNRNPUL1*, 58.3%; and 5 *MEF2D-BCL9*, 41.7%), and 14 (4.4%) had *Ph-like* fusion. Among *Ph-like* patients, 4 (1.3%) had *P2RY8-CRLF2*, whereas 10 (3.1%) had *Ph-like non-P2RY8-CRLF2* fusion (30% *ABL1*: 1 *TEL-ABL1* and 2 *NUP214-ABL1*; 30% *ABL2*: 3 *RCSD1-ABL2*; 20% *PDGFRB*: 2 *EBF1-PDGFRB*; and 20% *JAK2*: 1 *EBF1-JAK2* and 1 *PCM1-JAK2*). The remaining 199 (62.2%) patients had no detectable fusions and were classified as B-other in the current study. In total, 25.3% (*n* ═ 81) of patients were identified with novel fusion transcripts, which was significantly higher than the frequency of patients with classical fusions (12.5%, *n* ═ 40, *P* < 0.001).

### Characteristics of patients with individual fusions

In the whole cohort, as shown in Table 1, there were significant differences in age, WBC count, hemoglobin, platelet count, *IKZF1* deletion, and high-risk karyotype among fusion-defined groups (*P* ═ 0.015, <0.001, 0.0071, <0.001, 0.0022, and <0.001, respectively). Sex and complex karyotype tended to be statistically insignificant among fusions defined groups (*P* ═ 0.080 and 0.056, respectively).

**Table 1:** Characteristics of patients at diagnosis

**Variable**	**All**	***KMT2A* rearrangement**	***TCF3-PBX1***	***Ph-like* fusion**	***ZNF384* fusion**	***MEF2D* fusion**	**B-other**	***P* value**
Number of patients	320	26	14	10	55	12	199	
Age (y, median, range)	31.0 (15.0–65.0)	42.0 (17.0–64.0)	34.5 (17.0–56.0)	36.5 (19.0–59.0)	27.0 (16.0–62.0)	29.0 (17.0–60.0)	30.0 (15.0–65.0)	0.015
Males (%)	152 (47.5%)	7 (26.9%)	8 (57.1%)	9 (64.3%)	22 (40.0%)	8 (66.7%)	98 (49.2%)	0.080
WBC count (×10^9^/L, median, range)	8.6 (0.3–512.2)	48.0 (1.6–512.2)	11.9 (1.1–56.6)	35.7 (1.7–155.7)	6.6 (0.9–246.4)	12.2 (5.5–41.7)	7.7 (0.3–249.4)	<0.001
Hemoglobin (g/L) (median, range)	86.0 (31.0–165.0)	72.0 (41.0–148.0)	96.5 (48.0–149.0)	76.5 (46.0–129.0)	94.0 (40.0–154.0)	112.5 (68.0–162.0)	84.0 (31.0–165.0)	0.0071
Platelet count (×10^9^/L, median, range)	67.5 (0.1–510.0)	31.0 (3.0–99.0)	54.5 (13.0–308.0)	91.5 (15.0–203.0)	140.0 (12.0–368.0)	48.5 (15.0–317.0)	61.0 (0.1–510.0)	<0.001
*IKZF1* deletion (%) (*n* ═ 216)	58 (26.9%)	2 (2/16, 12.5%)	0 (0/9, 0%)	6 (6/8, 75.0%)	14 (14/34, 41.2%)	1 (1/9, 11.1%)	35 (35/140, 25.0%)	0.0022
Complex karyotype (%) (*n* ═ 241)	37 (15.4%)	4 (4/18, 22.2%)	2 (2/10, 20.0%)	2 (2/8, 25.0%)	1 (1/42, 2.4%)	1 (1/5, 20.0%)	27 (27/158, 17.1%)	0.056
High-risk karyotype (%) (*n* ═ 241)	53 (22.0%)	16 (16/18, 88.9%)	2 (2/10, 20.0%)	2 (2/8, 25.0%)	2 (2/42, 4.8%)	1 (1/5, 20.0%)	30 (30/158, 19.0%)	<0.001

Comparisons were further performed between patients with and without a certain type of fusion (Table S1). The most prominently significant comparisons appeared in *KMT2A* rearrangement, *Ph-like* and *ZNF384* fusion groups. *KMT2A* rearrangement was significantly related to older ages, higher WBC counts, lower hemoglobin contents, lower platelet counts, and higher frequency of high-risk karyotype (*P* <0.001, <0.001, ═0.015, <0.001, and <0.001, respectively); *Ph-like* fusion was significantly related to higher WBC counts and higher frequency of *IKZF1* deletion (*P* ═ 0.011 and *=* 0.0054, respectively); *ZNF384* fusion was significantly related to higher platelet counts and lower frequency of high-risk karyotype (*P* ═ < 0.001 and 0.0018, respectively). In addition, patients with *MEF2D* fusion had higher hemoglobin levels at diagnosis than *MEF2D*-negative patients (*P* ═ 0.0076). Patients with B-other had significantly more near-normal WBC counts and lower-than-normal platelet counts than those with fusion genes (*P* ═ 0.0035 and 0.013, respectively). Patients with *TCF3-PBX1* did not show statistically significant characteristics at diagnosis (all *P* ≥ 0.05).

### Impact of fusion types on CR achievement

Out of all 320 patients, 279 (87.2%) achieved CR after one course of induction therapy. The 1-course CR rate tended to be statistically significant among fusions defined groups (*P* ═ 0.084). Comparisons between patients with and without certain fusions were further performed (Table S2A). Statistical significance was observed only in *Ph-like* group, and patients with *Ph-like* fusion had a significantly lower 1-course CR achievement rate than those without *Ph-like* fusion (9/14 vs. 270/306, 64.3% vs. 88.2%, *P* ═ 0.023). Pairwise comparisons between *Ph-like* fusion and other fusion-defined subgroups were further performed. With the exception of a similar 1-course CR achievement rate to *TCF3-PBX1* (9/14 vs. 2/14, 64.3% vs. 85.7%, *P* ═ 0.39), *Ph-like* fusion had significantly lower 1-course CR achievement rate than that of other fusion-defined subgroups (all *P* < 0.05, Table S2B).

### Prognostic impact of fusion types on survival in the whole cohort

**Figure 3.**
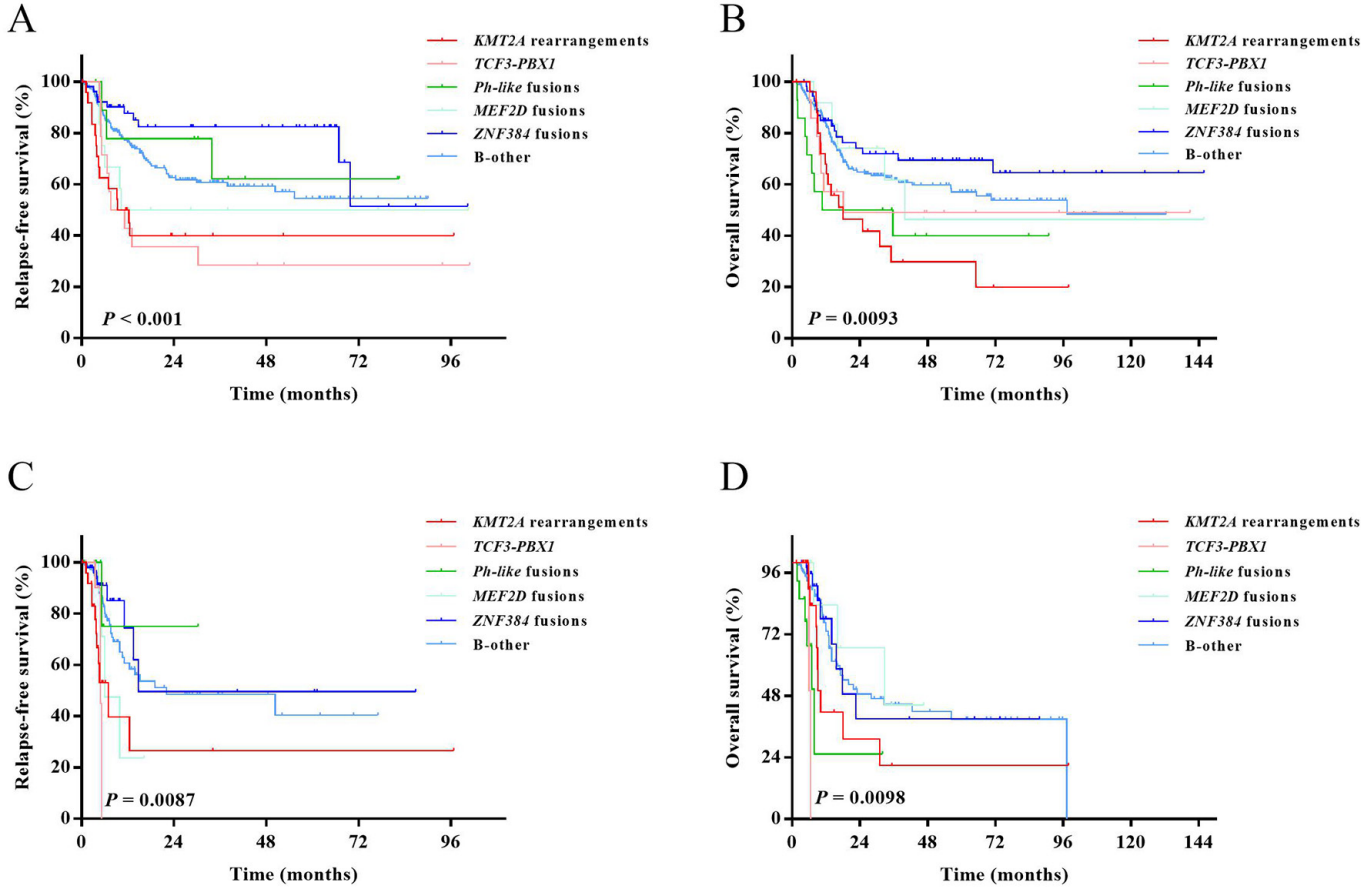
**RFS and OS in fusion-defined groups**. (A) RFS in the whole cohort; (B) OS in the whole cohort; (C) RFS of patients who received allo-HSCT and were censored at the time of transplantation; (D) OS of patients who received allo-HSCT and were censored at the time of transplantation. RFS: Relapse-free survival; OS: Overall survival; allo-HSCT: Allogeneic hematopoietic stem cell transplantation.

Both RFS and OS rates among the fusion-defined groups were statistically significant (*P* < 0.001 and *P* ═ 0.0093, respectively; Figure 3A and 3B).

Comparisons between patients with and without certain fusions were further performed (Table S2A). Patients with *KMT2A* rearrangement and *TCF3-PBX1* had significantly lower 3-year RFS rates (40% [95% CI: 20.4%–59.0%] vs. 62.3% [95% CI: 55.6%–68.3%], *P* ═ 0.0026; 28.6% [95% CI: 8.8%–52.4%] vs. 62.4% [95% CI: 55.8%–68.2%], *P* ═ 0.012, respectively), and patients with *ZNF384* fusions had a significantly higher 3-year RFS rate (82.4% [95% CI: 67.5%–90.9%] vs. 55.9% [95% CI: 48.7%–62.5%], *P* ═ 0.0062) than those without the corresponding fusions, respectively. *KMT2A* rearrangement also demonstrated a significantly lower 3-year OS rate (29.8% [95% CI: 12.2–49.9] vs. 62.2% [95% CI: 55.9–67.8], *P* ═ 0.0028), and *Ph-like* and *ZNF384* fusions individually tended to have a lower and higher 3-year OS rate (40.0% [95% CI: 14.5–64.7] vs. 60.4% [95% CI: 54.2–66.1], *P* ═ 0.082; 72.0% [95% CI: 57.2–82.4] vs. 56.8% [95% CI: 50.0–63.0], *P* ═ 0.054, respectively) than those without the corresponding fusions. Other comparisons between patients with and without a certain type of fusion gene were all statistically insignificant (all *P* ≥ 0.05).

### Prognostic impact of fusion types on survival in patients under chemotherapy treatment

Patients who received allo-HSCT were censored at the time of transplantation. Both RFS and OS rates among the fusion-defined groups were statistically significant (RFS, *P* ═ 0.0087; OS, *P* ═ 0.0098, Figure 3C and 3D).

Comparisons between patients with and without certain fusions were further performed (Table S2B). Patients with *KMT2A* rearrangement had a significantly lower 3-year RFS rate (26.5% [95% CI: 5.0–55.4] vs. 47.5 [95% CI: 35.5–58.6], *P* ═ 0.0011), and patients with *Ph-like* fusions had significantly lower OS rate (25.3% [95% CI: 1.4–64.1] vs. 40.9% [95% CI: 30.6%–51.0%], *P* ═ 0.0012) than patients without the corresponding fusions. Other comparisons between patients with and without a certain type of fusion gene were statistically insignificant (all *P* ≥ 0.05).

### Grouping patients based on fusion types

Based on the comparisons between patients with and without certain fusions, patients with *KMT2A* rearrangement and *ZNF384* fusions were individually related to prominently unfavorable and favorable outcomes. Therefore, *KMT2A* rearrangement and *ZNF384* fusion were designated as the references for high-risk and standard-risk groups, respectively. The other fusion-defined subgroups were compared with these two groups for further risk stratification (Tables S3A and S3B).

In the entire cohort, B-other had a significantly higher 3-year survival rate than *KMT2A* rearrangement in both RFS and OS (3-year RFS rate: 60.7% [95% CI: 52.4–68.1] vs. 40.0% [95% CI: 20.4–59.0], *P* ═ 0.0055; 3-year OS rate: 61.8% [95% CI: 53.9–68.7] vs. 29.8% [95% CI: 12.2–49.9], *P* ═ 0.0033, respectively). In patients under chemotherapy, B-other also had a significantly higher 3-year RFS rate than *KMT2A* rearrangement (48.4% [95% CI: 34.6–61.0] vs. 26.5% [95% CI: 5.0–55.4], *P* ═ 0.0021). In addition, *TCF3-PBX1* showed a lower 3-year RFS rate in the entire cohort (28.6% [95% CI: 8.8–52.4] vs. 82.4% [95% CI: 67.5–90.9], *P* < 0.001), and a lower 3-year RFS and OS rates in patients under chemotherapy than *ZNF384* fusion (3-year RFS rate: 0 vs. 49.6% [95% CI: 17.2–75.6], *P* ═ 0.032; 3-year OS rate: 0 vs. 39.0% [95% CI: 12.8–65.0], *P* ═ 0.010, respectively). *Ph-like* fusion also showed significantly lower 3-year OS rates than *ZNF384* fusions either in the entire cohort or in patients under chemotherapy (40.0% [95% CI: 14.5–64.7] vs. 72.0% [95% CI: 57.2–82.4], *P* ═ 0.018; 25.3% [95% CI: 1.4–64.1] vs. 39.0% [95% CI: 12.8–65.0], *P* ═ 0.0033, respectively). We observed a significantly lower 3-year RFS rate compared B-other to *ZNF384* fusions in the whole cohort (B-other vs. ZNF384 fusions, 60.7% [95% CI: 52.4%–68.1%] vs. 82.4% [95% CI: 67.5%–90.9%], *P* ═ 0.025). Other comparisons with *KMT2A* rearrangement and *ZNF384* fusion were all statistically insignificant (all *P* ≥ 0.05).

Based on the above-mentioned analysis, B-other had a significantly more favorable prognosis than *KMT2A* rearrangement, while *TCF3-PBX1* and *Ph-like* fusion had significantly more unfavorable prognosis than *ZNF384* fusion. However, there were no statistically significant differences of *MEF2D* fusion either with *KMT2A* rearrangement or *ZNF384* fusion, but, we still saw a tendency of lower 3-year RFS rate in the whole cohort compared with *ZNF384* fusion (50.0% [95% CI: 20.8–73.6] vs. 82.4% [95% CI: 67.5–90.9], *P* ═ 0.052).

As a result, we divided all patients into two groups. The first group was the fusion-defined standard-risk group (standard-risk group, *n* ═ 254, 79.4%) which included patients with *ZNF384* fusion and B-other. The second group was the fusion-defined high-risk group (high-risk group, *n* ═ 66, 20.6%) which included patients with *KMT2A* rearrangement, *TCF3-PBX1*, *Ph-like,* and *MEF2D* fusions.

**Figure 4.**
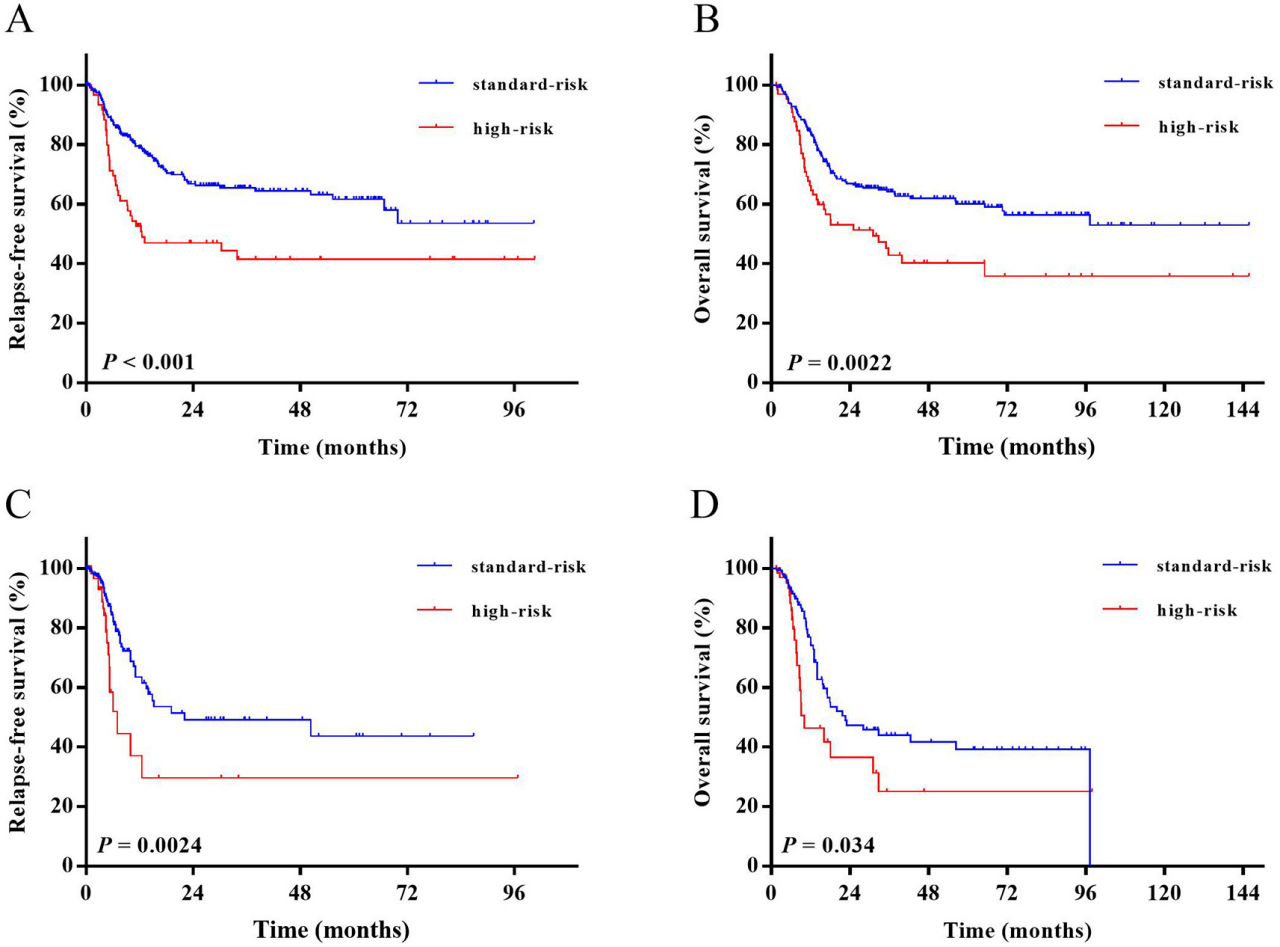
**RFS and OS in fusion-defined risk.** (A) RFS in the whole cohort; (B) OS in the whole cohort; (C) RFS of patients who received allo-HSCT and were censored at the time of transplantation; (D) OS of patients who received allo-HSCT and were censored at the time of transplantation. RFS: Relapse-free survival; OS: Overall survival; allo-HSCT: Allogeneic hematopoietic stem cell transplantation.

As shown in Figure 4A and 4B, the high-risk group had both significantly lower 3-year RFS and 3-year OS rates than standard-risk group (3-year RFS rate, 41.6% [95% CI: 28.2–54.5] vs. 65.4% [95% CI: 58.2–72.1], *P* < 0.001; 3-year OS rate, 42.8% [95% CI: 29.9–55.2] vs. 64.1% [95% CI: 57.4–70.1], *P* ═ 0.0022).

Survival functions were also performed when the cohort was censored at the time of transplantation (Figure 4C and 4D). High-risk group had both significantly lower 3-year RFS rate and 3-year OS rate than standard-risk group (3-year RFS rate, 29.7% [95% CI: 10.6–51.8] vs. 49.2% [95% CI: 36.4–60.8], *P* ═0.0024; 3-year OS rate, 25.0% [95% CI: 9.2–44.7] vs. 43.9% [95% CI: 32.3–55.0], *P* ═0.034).

### Univariate and multivariate analysis

In the whole cohort, in addition to fusion-defined high-risk group, age ≥ 40, WBC count ≥ 30 × 10^9^/L, platelet count < 60 × 10^9^/L, treatment with chemotherapy alone and not achieving CR within four weeks were significantly related to both lower RFS and OS rates (all *P* < 0.05), and high-risk karyotype was significantly related only to lower OS rate (*P* < 0.05), but not RFS rate. Gender, *IKZF1* deletion or not, MRD > 0.01% at remission and after first consolidation or not were all irrelevant to RFS and OS rates (all *P* ≥ 0.05, Table 2). The multivariate analysis showed that fusion-defined high-risk group, treating with chemotherapy alone, and not achieving CR within four weeks were independent poor prognostic factors for RFS. Additionally, ages 40 and up, treating with chemotherapy alone, and not achieving CR within four weeks were independent poor prognostic factors for OS (all *P* < 0.05, Table 3). Fusion-defined risk stratification was not significantly related to OS independently (*P* ═ 0.47).

**Table 2:** *P* values of univariate analysis in adult Ph-negative BCP-ALL in this cohort

**Variable**	**RFS**	**OS**
Fusion-defined risk (high-risk group vs. standard-risk group)	<0.001	0.0022
Age (>═ 40 vs. <40)	0.016	<0.001
Sex (Male vs. Female)	0.55	0.16
WBC count (×10^9^/L) (>═ 30 vs. <30)	<0.001	0.0040
Hemoglobin (g/L) (<═ 90 vs. >90)	0.75	0.85
Platelet count (×10^9^/L) (<60 vs. >═ 60)	0.0017	0.011
High-risk karyotype (yes vs. no) (*n* ═ 241)	0.22	0.032
*IKZF1* deletion (yes vs. no) (*n* ═ 216)	0.95	0.84
Treatment modality (chemotherapy alone vs. allo-HSCT)	<0.001	<0.001
Achieving CR after 1-course induction (no vs. yes)	<0.001	<0.001
MRD>0.01% at remission (yes vs. no) (*n* ═ 284)	0.43	0.32
MRD>0.01% after 1st consolidation (yes vs. no) (*n* ═ 270)	0.086	0.41

**Table 3:** Multivariate analysis of RFS and OS in adult Ph-negative BCP-ALL in this cohort

**Variable**	**RFS**		**OS**	
	**HR (95% CI)**	***P* value**	**HR (95% CI)**	***P* value**
Fusion-defined risk (high-risk group vs. standard-risk group)	2.35 (1.55–3.55)	<0.001		0.47
Age (>═ 40 vs. <40)		0.42	2.25 (1.49–3.39)	<0.001
WBC count (×10^9^/L) (>═ 30 vs. <30)		0.10		0.49
Platelet count (×10^9^/L) (>═ 60 vs. <60)		0.080		0.48
High-risk karyotype (*n* ═ 241)				0.37
Treatment modality (chemotherapy alone vs. allo-HSCT)	5.69 (3.80–8.50)	<0.001	3.31 (2.14–5.13)	<0.001
Achieving CR after 1-course induction (no vs. yes)	2.29 (1.25–4.19)	0.0070	4.96 (2.97–8.27)	<0.001

## Discussion

High-risk BCP-ALL patients had high relapse rates and poor outcomes when given a standard chemotherapy regimen. Exploring optimized chemotherapy, immunotherapy, or targeted small molecule inhibitors relied on a more complete identification of genomic profiles and more precise risk stratification based on molecular biology [27]. By performing RQ-PCR, 12.5% and 25.3% of adult Ph-negative BCP-ALL patients were individually identified as classical and novel fusion transcripts. Based on the results of survival analysis, patients were classified into two fusion-defined risk groups: the high-risk group including *KMT2A* rearrangement, *TCF3-PBX1*, *Ph-like,* and *MEF2D* fusions, and the standard-risk groups including *ZNF384* fusion and B-other.

In the current study, 37.8% of patients were successfully identified fusion transcripts, and two-thirds of them had novel fusion transcripts. The *ZNF384* fusion was most frequently detected in the entire cohort and had different fusion partners, of which *EP300-ZNF384* was most frequently detected. This result was consistent with what Yasuda et al. [28] reported from a single Japanese center. But reports from UKALLXII/E2993 B-ALL cohort and Australia showed a quite low incidence of *ZNF384* fusion [29, 30]. These may suggest a varied incidence of *ZNF384* fusion among different ethnic groups (higher presence in Asians compared to western races). As a continuation and complement, we obtained similar results in clinical characteristics and survival outcomes for *ZNF384* fusion with our previous study—with more near-normal clinical characteristics like significantly higher platelet counts, lower incidence of high-risk karyotype at diagnosis, and longer RFS period than those with no *ZNF384* fusion [13]. Although studies from western countries consistently classified *ZNF384* fusion into intermediate prognostic group due to lower satisfactory long-term survival than expected [29], a favorable prognosis was observed in both our previous and present cohorts.

The second most frequent fusion transcript was *KMT2A*-related rearrangement, with a similar incidence compared to reported studies in different races [29, 31, 32]. Patients with *KMT2A* rearrangement showed quite unfavorable clinical characteristics at diagnosis, which was consistent with findings of relevant studies reviewed by El Chaer et al. [33]. Similar to UKALLXII/E2993 B-ALL cohort [29], patients with *KMT2A*-rearrangement had an extremely adverse long-term survival both in the entire cohort and in patients undergoing chemotherapy. Furthermore, consistent with previous reports [34, 35], prominently low RFS rates along with poor prognosis due to early relapse were seen in *KMT2A*-rearranged patients in the current cohort.

*TCF3-PBX1* was recurrently detected in 4.4% of patients and the incidence was consistent with previous reports [29, 36]. Though not prominently characterized at diagnosis, *TCF3-PBX1*-positive patients had poor long-term prognosis in the current cohort. However, results from both UKALLXII/E2993 B-ALL cohort and a single Japanese center showed favorable outcomes in *TCF3-PBX1-*positive patients, even more favorable than *ZNF384* fusion [28, 29]. In addition, in version 2.2021 of the NCCN clinical practice guidelines, *TCF3-PBX1* has not been included in the high-risk group [26]. But remarkably, several previous studies, along with ours, showed that patients with *TCF3-PBX1* had significantly lower RFS rates than *TCF3-PBX1*-negative patients [13, 37–39]. Furthermore, previous studies showed remarkable efficacy of allo-HSCT in *TCF3-PBX1* patients [36, 38, 40]. In our current study, in patients under chemotherapy, *TCF3-PBX1* showed a quite low 3-year OS rate even lower than *KMT2A* rearrangement, but this low OS rate ceased to exist when this comparison with *KMT2A* rearrangement was performed in the whole cohort, which confirmed that allo-HSCT was a potential treatment for patients with *TCF3-PBX1*.

*MEF2D* fusion was detected in 3.8% of patients. In addition to significantly higher hemoglobin levels at diagnosis than negative patients, *MEF2D*-positive patients had no prominent clinical features or survival outcomes in the current cohort. Jeha et al. [41] categorized *MEF2D* fusions into unfavorable subtypes in a child ALL cohort for the low event-free survival rates. Taking *DUX4* as a reference, Paietta et al. classified *MEF2D* fusions into molecular intermediate risk group with a significantly lower 5-year RFS rate, but no statistical significance was observed in the 5-year OS rate. This is partly due to remarkable individualistic differences within the *MEF2D*-positive group [29]. In our current cohort, there were also no statistical significances in the comparisons between *MEF2D*-positive and -negative patients nor in comparisons with *KMT2A* rearrangement and *ZNF384* fusion. Although wide individual differences and a small sample size of *MEF2D* fusion made it difficult to show statistical significance, we still observed a poor prognostic trend in the long-term follow-up period from the survival curves. As a result, we categorized *MEF2D* into high-risk group.

Although the incidence of adult *Ph-like* BCP-ALL reached 20% to 30% among Caucasians [21], it was still less well established in Asian cohorts. The *Ph-like* fusion accounted for just 4.4% of the current cohort, which was similar to that in a recent report from Taiwan [32]. Although it is impossible to screen all *Ph-like* ALL fusions by PCR, we covered the common fusion types and fusion sites according to literatures and our RNAseq results. Therefore, *Ph-like* fusions in Chinese appear to be not as common as that in reports from western countries. Literature data have confirmed inferior outcomes in adult patients both with *Ph-like* ALL and the *CRLF2^+^* subset of *Ph-like* ALL [29, 42]. Consistent with previous reports, in the present cohort, *Ph-like* patients had a high frequency of *IKZF1* deletion, a high non-response rate to chemotherapy, and poor OS if only chemotherapy was given [22, 42]. However, *Ph-like* fusion reported in western reports with high relapse rates was not reflected in the present cohort [29]. Although *Ph-like* patients in the current study failed to enjoy favorable CR and OS rates, long-term RFS was promising anyway. This may suggest that once CR was achieved, favorable long-term survival was highly likely to be achieved in *Ph-like* patients. Moreover, in accordance with the report by Morak et al. [43], we found that approximately one-third of patients had detectable varied levels of *P2RY8-CRLF2* transcripts. Because the highest *P2RY8-CRLF2* level of patients with other fusion genes was 2.01%, we selected it as the cut-off value to define *P2RY8-CRLF2* positive. As a result, there were only four *P2RY8-CRLF2*-positive cases, and clinical characteristics and survival outcomes of *P2RY8-CRLF2* in adult BCP-ALL needed to be explored further.

Based on the results of risk stratification, multivariate analysis showed that the fusion-defined high-risk group was independently related to poor RFS but not OS. Contrary to fusion-defined risk, subjects aged 40 years or older was an independent poor prognostic factor for OS but not RFS, which suggested that compared to leukemia itself, patients’ physical status was a key factor for survival. This was in accordance with the classical prognostic factor for subjects greater than 35 years for adult ALL [44, 45]. Furthermore, it implied that risk stratification should be performed based on multiple factors.

This study had several special features. First, consecutive adult cases newly diagnosed with Ph-negative BCP-ALL were included, so incidence and survival rates were convincing. Second, fusion transcript screening and risk stratification establishment were based on RQ-PCR, which was simple, rapid, economical, and widely applicable in a large-scale clinical practice. However, several limitations still existed in this study. First, *IGH*-related fusions, such as *IGH-CRLF2* and *DUX4-IGH*, were not covered in this study, for highly variable sequences and positions of breakpoints within *IGH* made it difficult to test by RQ-PCR method. Second, as this was a retrospective study, treatment regimens may not be completely implemented. These limitations strained further classification in B-other group and had an impact on more precise risk stratification. Although implementation of transcriptome sequencing (RNA-seq) could lead to a wider discovery of novel molecular entities, it would still take longer for this to be generally used in clinical practice.

By performing RQ-PCR, 37.8% adult Ph-negative BCP-ALL patients were identified fusion transcripts and the spectrum of fusion genes in Chinese cohort was distinct from that in reports from western countries. The types of fusion transcripts are relevant to clinical features and outcomes. The novel fusions, ZNF384 was defined as standard risk, and Ph-like and MEF2D fusions were defined as high risk. More multicenter and prospective clinical cohort studies are required to incorporate fusion transcripts into precise risk stratification system to guide optimized therapy.

## Supplemental Data

**Table S1:** *P* values for comparisons between patients with and without certain fusions on patients’ characteristics at diagnosis

**Sample 1-Sample 2**	**Age**	**WBC**	**HB**	**PLT**	**IKZF1**	**High-risk karyotype**
*KMT2A* rearrangements vs. non-*KMT2A*-rearrangements	**<** **0.001**	**<** **0.001**	**0.015**	**<** **0.001**	0.25	**<** **0.001**
*TCF3-PBX1* vs. non-*TCF3-PBX1*	0.80	0.97	0.16	0.53	0.12	1.0
*Ph-like* fusions vs. non-*Ph-like* fusions	0.19	**0.011**	0.56	0.44	**0.0054**	1.0
*ZNF384* fusions vs. non-*ZNF384* fusions	0.29	0.14	0.26	**<** **0.001**	0.056	**0.0018**
*MEF2D* fusions vs. non-*MEF2D* fusions	0.77	0.45	**0.0076**	0.70	0.45	1.0
B-other vs. non-B-other	0.10	**0.0035**	0.37	**0.013**	0.43	0.14

**Table S2A:** *P* values for comparisons between patients with and without certain fusions on 1-course CR achievement rate, survival of RFS and OS in the whole cohort

**Sample 1-Sample 2**	**1-course CR Achievement**	**RFS**	**OS**
*KMT2A* rearrangements vs. non-*KMT2A*-rearrangements	0.55	**0.0026**	**0.0028**
*TCF3-PBX1* vs. non-*TCF3-PBX1*	0.70	**0.012**	0.41
*Ph-like* fusions vs. non-*Ph-like* fusions	**0.023**	0.55	**0.082**
*ZNF384* fusions vs. non-*ZNF384* fusions	0.27	**0.0062**	**0.054**
*MEF2D* fusions vs. non-*MEF2D* fusions	0.38	0.34	0.85
B-other vs. non-B-other	0.49	0.66	0.37

**Table S2B:** *P* values for pairwise comparisons with *Ph-like* fusions of 1-course CR achievement rate in the whole cohort

**Fusion-defined subgroups**	**Ph-like fusions**
*KMT2A* rearrangements	**0.039**
*TCF3-PBX1*	0.39
*ZNF384* fusions	**0.014**
*MEF2D* fusions	**0.042**
B-other	**0.046**

**Table S2C:** *P* values for comparisons between patients with and without certain fusions on survival of RFS and OS in the whole cohort censored at the time of transplantation

**Sample 1-Sample 2**	**RFS**	**OS**
*KMT2A* rearrangements vs. non-*KMT2A*-rearrangements	**0.0011**	0.18
*TCF3-PBX1* vs. non-*TCF3-PBX1*	0.15	0.21
*Ph-like* fusions vs. non-*Ph-like* fusions	0.54	**0.0012**
*ZNF384* fusions vs. non-*ZNF384* fusions	0.14	0.39
*MEF2D* fusions vs. non-*MEF2D* fusions	0.36	0.44
B-other vs. non-B-other	0.29	0.29

**Table S3A:** *P* values for pairwise comparisons with *KMT2A* rearrangements and *ZNF384* fusions on survival of RFS and OS in the whole cohort

	**RFS**		**OS**	
**Fusion-defined subgroups**	***KMT2A* rearrangements**	***ZNF384* fusions**	***KMT2A* rearrangements**	***ZNF384* fusions**
*TCF3-PBX1*	0.96	**<** **0.001**	0.47	0.12
*Ph-like* fusions	0.12	0.64	0.84	**0.018**
*MEF2D* fusions	0.49	**0.052**	0.094	0.48
B-other	**0.0055**	**0.025**	**0.0033**	0.18

**Table S3B:** *P* values for pairwise comparisons with *KMT2A* rearrangements and *ZNF384* fusions on survival of RFS and OS in the whole cohort censored at the time of transplantation

	**RFS**		**OS**	
**Fusion-defined subgroups**	***KMT2A* rearrangements**	***ZNF384* fusions**	***KMT2A* rearrangements**	***ZNF384* fusions**
*TCF3-PBX1*	0.55	**0.032**	**0.038**	**0.010**
*Ph-like* fusions	0.10	0.86	0.12	**0.0033**
*MEF2D* fusions	0.33	0.15	0.16	0.61
B-other	**0.0021**	0.33	0.15	0.69
